# Nanopore 16S-Full Length and ITS Sequencing for Microbiota Identification in Intra-Abdominal Infections

**DOI:** 10.3390/diagnostics15172257

**Published:** 2025-09-06

**Authors:** Jian-Jhou Liao, Yong-Sian Chen, Hui-Chen Lin, Yi-Ju Chen, Kuo-Lung Lai, Yan-Chiao Mao, Po-Yu Liu, Han-Ni Chuang

**Affiliations:** 1 Division of General Surgery, Department of Surgery, Taichung Veterans General Hospital, Taichung 40705, Taiwan; ljjhou@gmail.com (J.-J.L.); newmotlesimilu@gmail.com (Y.-S.C.); joaquinmaxo@yahoo.com.tw (H.-C.L.); chenyiju5668@gmail.com (Y.-J.C.); 2 Institute of Medicine, Chung Shan Medical University, Taichung 40201, Taiwan; 3 Division of Allergy, Immunology and Rheumatology, Taichung Veterans General Hospital, Taichung 40705, Taiwan; kllaichiayi@yahoo.com.tw; 4 Division of Clinical Toxicology, Department of Emergency Medicine, Taichung Veterans General Hospital, Taichung 40705, Taiwan; doc1385e@gmail.com; 5 Division of Infectious Diseases, Department of Internal Medicine, Taichung Veterans General Hospital, Taichung 40705, Taiwan; liupoyu@gmail.com; 6 Genomic Center for Infectious Diseases, Taichung Veterans General Hospital, Taichung 40705, Taiwan; 7 Rong Hsing Translational Medicine Research Center, National Chung Hsing University, Taichung 402202, Taiwan; 8 Department of Post-Baccalaureate Medicine, College of Medicine, National Chung Hsing University, Taichung 402202, Taiwan; 9 Department of Medical Research, Taichung Veterans General Hospital, Taichung 40705, Taiwan

**Keywords:** intra-abdominal infections, nanopore sequencing, ONT sequencing, 16S, ITS

## Abstract

**Background/Objectives**: Intra-abdominal infections (IAIs) constitute significant clinical challenges that can rapidly progress to life-threatening conditions if not promptly diagnosed and treated. Traditional pathogen identification methodologies, predominantly culture-based, frequently necessitate extended turnaround times (TATs) and exhibit limitations in detecting polymicrobial or anaerobic infections. **Methods**: We implemented Oxford Nanopore Technology (ONT) sequencing to analyze the microbiota in patients with IAIs at Taichung Veterans General Hospital. The study cohort comprised sixteen patients with IAIs. Following specimen collection, DNA extraction was performed, and then full-length 16S rRNA and ITS region amplification and subsequent ONT sequencing were conducted. **Results**: Conventional clinical culture-based methodologies detected pathogens in 13 patients. Among the 14 successfully sequenced specimens, ONT sequencing elucidated a diverse spectrum of bacteria and fungi, with read counts ranging from 375 to 19,716. Polymicrobial and anaerobe-enriched communities were predominantly observed in lower gastrointestinal tract infections, specifically colonic or small bowel perforations, whereas upper gastrointestinal perforations, including those of the stomach or duodenum, were frequently dominated by *Streptococcus*, *Granulicatella*, or *Candida* species. The sequencing identified pathogens concordant with culture results, including *Escherichia coli*, *Enterococcus*, and *Candida albicans*. In addition, anaerobic or low-abundance taxa were exclusively identifiable through sequencing methodologies. **Conclusions**: ONT sequencing facilitated results within up to 24 h and successfully detected pathogens in culture-negative cases. These findings underscore the utility of ONT sequencing as an expeditious and comprehensive diagnostic modality for IAIs.

## 1. Introduction

Intra-abdominal infections (IAIs) usually arise after a breach in the intrinsic mucosal defense barrier that allows normal bowel flora to inoculate the abdominal cavity. The precise microbiological spectrum depends on the precise gastrointestinal source (i.e., small versus large bowel) [1]. IAIs typically result from inflammation or disruptions in the gastrointestinal tract, such as peritonitis, cholangitis, diverticulitis, pancreatitis, abdominal abscesses, intestinal perforations, abdominal trauma, and pelvic inflammatory disease [2]. IAIs are the second most common cause of infectious morbidity and mortality in the ICU (Intensive Care Unit), following pneumonia [2]. Mortality rates associated with secondary peritonitis with severe sepsis or septic shock have reported an average mortality of approximately 30% [3]. IAIs represent a significant clinical challenge, ranging from localized infections such as appendicitis to life-threatening conditions like diffuse peritonitis [4]. Conventional pathogen detection methods, primarily based on culture techniques, often require 24–72 h or longer to yield definitive results. This time lag is particularly problematic in the management of IAI, where rapid initiation of targeted antimicrobial therapy is critical—studies have shown that each hour of delay in administering appropriate antibiotics in septic patients can increase mortality risk by up to 10% [5]. While colonic flora consists of approximately 400 species, an average of only 4 to 6 species are generally recovered from these IAIs. The dominant isolates in most series are *Bacteroides fragilis* and *E. coli.* IAIs are typically polymicrobial infections, characterized by the presence of multiple pathogenic species, which pose a significant challenge in clinical settings. These organisms can be isolated in clinical laboratories, and a number of studies have been undertaken to better understand the ability of specific organisms to cause infection and survive based upon their virulence factors and capacity to adapt to new environmental conditions [6]. For critically ill patients, if antibiotics must be initiated before collecting samples, it is best to collect the samples within a few hours after starting antibiotics, as these culture results are more meaningful than those collected after prolonged antibiotic exposure.

Nanopore sequencing (Oxford Nanopore Technologies, Oxford, UK) is a third-generation, single-molecule sequencing technology that generates long sequence read lengths [7]. This technology enables real-time detection of single-molecule sequences and is characterized by its culture-free and rapid processing capabilities [8]. It minimizes bias during library construction, as seen with PCR [8].

Previously, ONT sequencing was used to identify fungal species in dogs in real-time [9], and has been successfully applied to human infectious diseases, including pathogen identification from cell-free DNA (cfDNA) in body fluids [10], as well as the direct detection of bacterial and fungal pathogens in clinical samples such as urine and respiratory secretions [11,12], and rapid profiling of bacterial communities in sterile body fluids, peritoneal dialysis effluent, sepsis blood cultures, and abscess material [13,14,15]. These studies highlight its potential for detecting pathogens in conditions highly analogous to IAIs. In this study, we want to utilize ONT sequencing to characterize the microbiota in patients with intra-abdominal infections.

## 2. Materials and Methods

### 2.1. Patient Information

This study was approved by the Institutional Review Board of Taichung Veterans General Hospital (IRB No. CE20004B), and written informed consent was obtained from all the participants prior to enrollment. The study was conducted in accordance with the Declaration of Helsinki. Patients aged ≥18 years with clinical diagnosed with intra-abdominal infections, including, but not limited to, complicated intra-abdominal infections and post-traumatic osteomyelitis, and admitted to Taichung Veterans General Hospital between January 2023 and January 2024 were enrolled. The exclusion criteria were pregnancy and human immunodeficiency virus (HIV) infection. Data collected included demographic information (age and gender), clinical manifestations, and management details, including surgical indications, timing, and types of surgical procedures performed.

### 2.2. Sample Collected and Processing

According to the procedures of the Center of Pathology & Clinical Medical Laboratory at Taichung Veterans General Hospital, specimens were collected as part of routine microbiological workups, including ascites, peritoneal drainage (PD) fluid and surgical debridement specimens. All the samples were stored at 4 °C and transferred to the Precision Medicine Laboratory for DNA extraction within 24 h. The IAI sample was centrifuged for 15 min at high speed to pellet cells. The pellet cell suspension and lytic enzyme solution were mixed and incubated for 30 m at 37 °C. The remaining procedure of the extraction was performed in accordance with the instructions provided in the Gentra puregene blood kit and Gentra Puregene Yeast/Bact. Kit (Qiagen, Hilden, Germany, cat. 158845/1042607). The extracted DNA was stored at −80 °C. The IAI specimen of biopsy microbial cultures (aerobic and anaerobic) were performed during surgical debridement. Bacteria were identified using the VITEK 2 system (BioMérieux Inc., Durham, NC, USA). Culture sampling was performed as previously described [16].

### 2.3. Clinical Identification

Bacterial and fungal cultures were performed on specimens obtained from surgical debridement. Species identification was carried out using the VITEK 2 system (BioMérieux Inc., Durham, NC, USA).

### 2.4. DNA Library Preparation and ONTsequence

In the laboratory, 0.5 μg of extracted DNA was amplified using 16S and ITS primers. The lengths of the 16S and ITS PCR products were 1500 bp and 400 to 800 bp, respectively. The amplified products were packaged into the library for the Nanopore system. DNA libraries were prepared according to the manufacturer’s instructions, using the ligation sequencing kit (SQK-LSK109) and the native barcoding kit (EXP-NBD104), including the optimized DNA sequence of the KAPA Hyper Prep Kit. The MinION (Oxford Nanopore Technologies, Oxford, UK) flow cell preparation and sample loading were conducted according to the SQK-LSK109 protocol using a 75 μL DNA library. Equal amounts of amplicons per sample were pooled, and the library was further processed following the manufacturer’s instructions. The sequencing mixture was added into the R9.4.1 or R10 flow cell for 48–72 h. During the ONT sequencing, the FAST5 files from MinION were converted to FASTQ files using MinKNOW (Oxford Nanopore Technologies) version 5.0.5 with a FAST model and a Q-score ≥ 7.

### 2.5. Bioinformatics Analysis

For the identification of microbiota at the genus level, fastq files containing full-length 16S rRNA and ITS region amplicons were analyzed with EPI2ME Labs wf-16s workflow. The sample was classified using the NCBI 16s_18s_28s_ITS dataset, read length 400 to 2000 bp. The exclusion criteria for single nanopore reads were an alignment count accuracy <80%.

## 3. Results

### 3.1. Clinical Characteristics of Patients with Intra-Abdominal Infections (IAIs)

A total of 16 patients diagnosed with IAIs were enrolled in this study. Their demographic characteristics are summarized in Table 1. There were eight males and eight females, with a median age of 60.5 years (range from 27 to 84 years old). The patients had conditions such as acute appendicitis (*n* = 2); gastrointestinal perforations such as small bowel, colon, gastric, or duodenal perforation (*n* = 12); small bowel obstruction (*n* = 1); and non-occlusive mesenteric infarction (*n* = 1). Median hsCRP level at presentation was 7.3 mg/dL (range, 0.43–41.5 mg/dL), while median WBC count was 6930/μL (range, 2200–22,240/μL). All the patients received empiric antimicrobial therapy, primarily involving broad-spectrum beta-lactams such as piperacillin, azobactam, ceftriaxone, cefepime, or ertapenem, in combination with metronidazole or antifungal agents, such as micafungin or anidulafungin, when fungal infection was suspected (Table 1).

### 3.2. Comparison of Conventional Clinical Culture and ONT Sequencing

**Figure 1:**
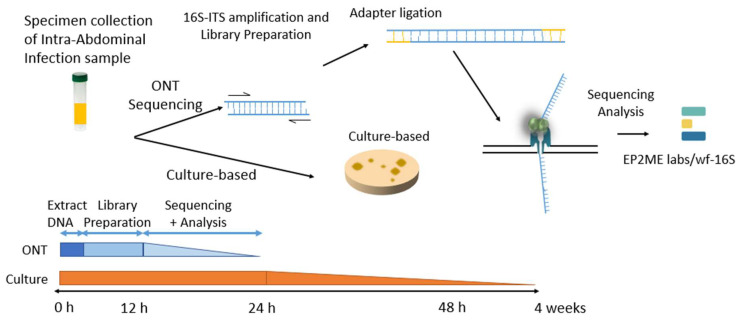
Comparison of ONT sequencing and conventional culture-based workflows for intra-abdominal infection diagnostics.

To evaluate the diagnostic timeline and resolution of different pathogen identification methods in IAIs, we compared the diagnostic workflows and turnaround times of conventional culture-based methods with those of ONT sequencing. The ONT sequencing workflow included 16S-ITS region amplification, library preparation, adapter ligation, and real-time sequencing, followed by taxonomic classification using the EPI2ME wf-16S pipeline. ONT sequencing allows DNA extraction and library preparation within 12 h, followed by sequencing and real-time microbial annotation, thereby providing a substantially faster turnaround time. Conventional clinical diagnosis relies on microbial culture for pathogen identification, which typically requires more than 24 h and may extend up to 4 weeks to complete. This is significantly faster than conventional cultures, which may take up to four weeks (Figure 1).

Clinical specimens collected and centrifuged to extract total DNA from intra-abdominal infections were processed using the ONT sequence and clinical culture-based approach. The nanopore-based approach includes 16S full length and ITS region amplification, library preparation with adapter ligation, followed by real-time sequencing and taxonomic analysis using the EP2ME labs/wf-16s workflow. In contrast, the conventional culture-based method involves sample plating and incubation for microbial growth and identification. The timeline at the bottom shows that the nanopore-based workflow can yield results within 12–24 h, whereas culture-based methods may take up to 4 weeks to complete.

### 3.3. ONT Sequencing Results and Diversity Metrics in IAI Patients

In addition, we used ONT sequencing to detect microbiota in IAI patient samples. Initially, all IAI patient samples underwent PCR amplification targeting the full-length 16S rRNA and ITS regions. However, IAI-06 and IAI-08 did not have PCR products, and therefore these samples were not subjected to ONT sequencing. We sequenced 14 samples, and the ONT sequencing identified total read counts ranging from 375 to 19,716 reads. The Fisher’s alpha diversity index varied between 7.44 and 46.88, with IAI-16 demonstrating the highest diversity and IAI-07 the lowest. The Inverse Simpson’s Index ranged from 1.22 to 6.13 across samples. Richness, defined as the number of observed species, was highest in IAI-04 (128 species) and lowest in IAI-16 (11 species). The Shannon diversity index values ranged from 1.00 to 2.18, with IAI-03 exhibiting the highest diversity and IAI-16 the lowest. The Pielou’s evenness index ranged from 0.14 to 0.51, indicating considerable variability in community evenness among samples; IAI-03 exhibited the highest evenness, while IAI-01 showed the lowest. In terms of bacterial abundance, IAI-04 had the highest bacterial count (19,676 reads), while IAI-01 showed the lowest (420 reads). Detection of eukaryotic sequences (primarily Candida species) varied among samples, with eukaryotes present in 11 of 14 sequenced samples but absent in IAI-02, IAI-03, and IAI-04 (Table 2). ONT sequencing effectively captures the full range of microbial diversity in IAI samples, providing insights that may inform more tailored antibiotic therapy and improve clinical outcomes.

### 3.4. Pathogen Detection by Conventional Culture and ONT Sequencing

Our cohort consisted of 14 patients with IAIs for ONT sequencing. In 18.8% of IAI patients (IAI-6, IAI-10, and IAI-14), no pathogens were identified through conventional clinical culture, including both ordinary and anaerobic systems (Table 3). Single pathogens in IAIs accounted for samples including IAI-01, IAI-05, IAI-08, IAI-09, IAI-13, IAI-15, and IAI-16, and the isolated organisms were *E. coli*, *Viridans streptococci*, *Klebsiella pneumoniae*, *Staphylococcus aureus*, and *C. albicans* Additionally, polymicrobial infections involving multiple bacteria species were detected in patients of IAI-03, IAI-07, and IAI-12. The isolated organisms in these IAI patients (IAI-02, IAI-03, and IAI-04) included *E. coli*, *Finegoldia magna*, *Pavimonas micra*, and *Veillonella dispar*, among others (Table 3).

Comparisons between conventional culture results and ONT sequencing data, among the 14 successfully sequenced samples, demonstrated that ONT sequencing detected pathogens concordant with culture results in the majority of cases. Moreover, *E. coli* was detected by both methods in IAI-01, IAI-02, and IAI-11, though its relative abundance varied from 0.21% to 7.36%. Similarly, *Enterococcus* species were identified by both culture and sequencing in IAI-03 and IAI-07. In samples where *C. albicans* was cultured (IAI-05, IAI-15, and IAI-16), ONT sequencing also confirmed its presence, with read proportions ranging from 8.91% to 86.05%.

Some discrepancies were observed between the two approaches. In IAI-04, *Streptococcus* was identified as the dominant organism by both culture and sequencing (61.89% by sequencing). However, additional genera such as *Veillonella*, *Prevotella*, *Oribacterium*, *Granulicatella*, and so on were also detected by ONT sequencing at a relative abundance of 10%, 7.62%, 2.63%, and 2.38%, while Lactobacillus was identified at a much lower abundance (0.72%) (Table 3). ONT sequencing demonstrated strong concordance with traditional culture for major pathogens, while also identifying additional minor or unculturable organisms not captured by conventional methods. These findings highlight the potential of ONT sequencing to provide a more comprehensive and rapid microbial profile in the diagnosis of intra-abdominal infections.

### 3.5. Association Between Microbial Composition Identified by ONT Sequencing and Clinical Phenotypes in IAI Patients

To evaluate the relationship between microbial and clinical phenotypes in IAI patients, genus-level taxonomic profiles obtained from ONT sequencing were compared with patients’ underlying disease types and infection presentations. In cases of acute appendicitis (IAI-01 and IAI-02), microbial profiles were relatively low in diversity, with dominant genera including *Sinocapsa* and *Streptococcus* (Table 4). Both patients exhibited mild clinical inflammation (hsCRP ≤ 1 mg/dL) and underwent laparoscopic appendectomy. Similarly, gastric or duodenal ulcer perforations (IAI-04, IAI-05, IAI-11, and IAI-15) showed varying degrees of microbial complexity. Notably, *Streptococcus* and *Bacteroides* were highly dominant in IAI-04 (61.89%) and IAI-11 (68.92%), both of which presented with septic shock or high inflammatory markers (hsCRP > 30 mg/dL). In contrast, *Candida* predominated in IAI-05 and IAI-15, with read proportions of 56.43% and 86.05% (Table 1 and Table 4), respectively. Patients with bowel perforations or colonic involvement (IAI-03, IAI-07, and IAI-12) exhibited highly polymicrobial profiles. These samples featured combinations of anaerobic genera such as *Prevotella*, *Fusobacterium, Parvimonas*, *Dialister*, and *Veillonella* (Table 4), consistent with translocation from the lower gastrointestinal tract. These patients also had moderate to high hsCRP levels and underwent extensive surgical interventions (e.g., subtotal colectomy or multi-segment resection).

In contrast, gastric ulcer with abscess (IAI-10) showed a broad bacterial profile including *Streptococcus*, *Granulicatella*, *Rothia*, and *Neisseria*, suggesting oral flora translocation (Table 4). However, no pathogen was identified by conventional culture in this case. (Table 3).

Overall, genus-level microbial patterns appeared to correspond with the anatomical site of infection and clinical severity. Upper gastrointestinal perforations were associated with dominance of *Streptococcus*, *Granulicatella*, or *Candida*, while lower GI or polymicrobial perforations displayed complex anaerobic communities. These findings suggest that microbial signatures obtained via ONT sequencing may reflect the source of infection and pathophysiological processes in IAI. It should provide a concise and precise description of the experimental results and their interpretation, as well as the experimental conclusions that can be drawn.

## 4. Discussion

The application of ONT sequencing technology for the detection of pathogens in IAIs represents a significant advancement over traditional culture-based diagnostic methods. Conventional clinical cultures, while reliable for many infections, often fail to detect polymicrobial or fastidious organisms due to their dependence on the ability of pathogens to grow under laboratory conditions. This limitation results in diagnostic delays and potentially inappropriate antimicrobial therapy, particularly critical in the context of IAIs where timely intervention significantly impacts patient outcomes [17,18].

In this study, we used ONT sequencing, which is a culture-independent approach, allowing direct insights into the microbial composition of clinical samples [19]. By amplifying 16S rRNA and ITS regions and sequencing them using nanopore technology, it was possible to identify a wide range of bacteria and fungi within a clinically relevant timeframe, often within 24 h. This rapid turnaround time is a stark improvement over traditional methods, which typically require several days to weeks for complete pathogen identification [19].

Our findings are consistent with recent studies that have demonstrated the clinical utility of ONT in infectious disease diagnostics. For example, Gu et al. applied metagenomic sequencing of infected body fluids and reported the ability to identify pathogens in real time [10]. Lao et al. further demonstrated that Nanopore 16S sequencing could directly identify bacteria from sterile body fluids, including peritoneal samples [12]. Harris et al. used ONT sequencing on blood culture isolates from ICU patients with sepsis, achieving rapid pathogen identification and antimicrobial susceptibility prediction [13]. Zhao et al. evaluated Nanopore sequencing of infectious fluids, including abscess material, and demonstrated high concordance with conventional culture [14]. Taken together, these studies support our observations and indicate that ONT sequencing provides clinically relevant results in contexts highly analogous to IAIs.

Despite its numerous advantages, ONT sequencing in clinical diagnostics also presents challenges. First, the relatively high error rate compared with short-read sequencing platforms can complicate species-level classification and antimicrobial resistance gene detection [20,21]. Second, PCR amplification of 16S rRNA and ITS regions, while useful for sensitivity, introduces potential amplification bias and may underrepresent certain taxa. Third, data interpretation requires substantial bioinformatics expertise, particularly when distinguishing true pathogens from background flora or contaminants. In conclusion, ONT sequencing represents a transformative tool for the diagnosis of IAIs, offering new avenues to improve patient care, reduce healthcare costs, and combat antimicrobial resistance.

### Limitation

This study has several limitations. First, the sample size was relatively small and was derived from a single center, which may limit the generalizability of our findings. Second, the lack of parallel quantitative microbiology or metagenomics confirmation precluded strain-level or resistance gene analysis, which could further inform treatment decisions.

Importantly, variation in sample collection and handling protocols may have influenced microbial detection. Delays in processing or oxygen exposure during transport could have disproportionately affected the recovery of strict anaerobes or fastidious organisms, thereby contributing to discrepancies between sequencing and culture-based results. Despite these limitations, the use of ONT sequencing provided valuable, culture-independent insights into the microbial landscape of intra-abdominal infections and revealed patterns not captured by conventional methods.

## 5. Conclusions

In this study, ONT sequencing was shown to provide culture-independent insights into the microbial communities of intra-abdominal infections within a clinically relevant timeframe of 24 h. The sequencing results were largely consistent with conventional culture findings and additionally revealed organisms that are typically difficult to recover by culture. These observations suggest that ONT sequencing may serve as a valuable complementary tool to traditional methods, particularly in polymicrobial infections of the lower gastrointestinal tract. However, further studies with larger cohorts are required to comprehensively evaluate its reproducibility, sensitivity, and specificity before its broader clinical application can be established. The ONT sequencing worked especially well for infections involving different types of bacteria. ONT sequencing can help clinic diagnose infections faster, leading to quicker and more effective treatment for patients.

## Figures and Tables

**Table 1:** Clinical characteristics of patients with intra-abdominal infection. The clinical characteristics of patients with IAI, including age, infection phenotype, operation type, inflammatory markers (hsCRP, WBC), and antibiotic treatments.

Sample	Gender	Age	Phenotype	hsCRP (mg/dL)	WBC Count	Operation	Antibiotic
1	M	27	Acute appendicitis	0.43	4960	laparoscopic appendectomy	Amoxicillin, Metronidazole
2	F	66	Acute appendicitis	1	12,130	laparoscopic appendectomy	Flomoxef
3	F	53	S-colon perforation with fecal peritonitis	-	6930	Hartmann’s procedure (sigmoidectomy + end D-colostomy)	Piperacillin, Ceftriaxone, Flomoxef
4	F	44	Acute gastric ulcer with perforation Septic shock	33.8	8030	simple suture with omental patch and round ligament patch	Fluconazole, Flomoxef, Anidulafungin, Ertapenem, Micafungin
5	F	84	Duodenal perforated ulcer	1.4	2200	laparoscopic duodenorrhaphy	Ivermectin, Doripenem, Amphotericin B liposomal, Vancomycin, Amoxicillin, Flomoxef
6	M	58	Small bowel obstruction, volvulus-related	1.3	6780	volvulus s/p reduction operation	Metronidazole, Amoxicillin
7	F	52	perforation of Jejunum Ileum adhesion to T-colon	13.4	15,840	resection of small bowel with anastomosis and Repair of colon	Ampicillin, Ceftriaxone, Anidulafungin, Flomoxef, Piperacillin
8	M	84	Non-occlusive mesentery infarction Septic shock	3.7	15,800	small intestine resection (420 cm) and end-jejunostomy	Tigecycline, Cefepime, Flomoxef, Ertapenem, Anidulafungin, Micafungin
9	F	76	Gastric ulcer perforation, type III	3.7	2770	simple closure with round ligament patch	Oxacillin, Tatumcef, Metronidazole, Micafungin, Ertapenem
10	F	46	Gastric ulcer with perforation, antrum, anterior wall/ intra-peritoneal abscess	7.3	6110	simple closure with round ligament patch	Ampicillin, Sulbactam, Micafungin, Ertapenem
11	M	78	Duodenal perforation	41.5	4800	primary repair of perforation and Foley sump drain drainage; debridement of retroperitoneum	Vancomycin, Flomoxef, Gentamicin, Piperacillin, Tazobactam, Piperacillin, Anidulafungin
12	M	41	Bowel perforation	11.5	19,150	small bowel resection, subtotal colectomy (with rectal stump remain); end-jejunostomy	Metronidazole, Cefepime, Anidulafungin, Amoxicillin, Flomoxef, Cefoperazone, Sulbactam
13	F	69	Perforation over gastrojejunostomy with generalized peritonitis	22.8	8720	take down of the gastrojejunostomy and reconstruction of a gastrojejunostomy	Ceftriaxone, Micafungin, Ertapenem
14	M	60	Gastric ulcer perforation, type III	0.7	5770	hemigastrectomy + B-II reconstruction (isoperistaltic, antecolic)	Micafungin, Vancomycin, Ertapenem, Cefuroxime
15	M	79	Duodenal ulcer perforation	2.1	22,240	laparoscopic duodenorrhaphy	Fluconazole, Micafungin, Ceftriaxone, Ertapenem
16	M	46	Perforated gastric ulcer	2.8	9660	primary closure with omentum patch	Amoxicillin, Ampicillin, Sulbactam, Vancomycin, Micafungin, Ertapenem

M, male; F, female.

**Table 2:** Alpha diversity indices and summary nanopore output total sequence reads for microbiota of specimens from intra-abdominal infections patients.

	1	2	3	4	5	7	9	10	11	12	13	14	15	16
Total counts	5109	16,235	8555	19,716	10,420	15,827	11,654	15,171	15,139	16,195	11,429	1094	3895	375
Fisher’s alpha	38.27	29.46	33.54	28.43	7.94	7.44	7.8	14.86	14.87	14.7	7.82	27.88	19.41	46.88
Inverse Simpson’s index	6.13	1.9	1.22	1.67	1.39	1.49	1.65	1.75	2.1	1.67	1.74	2.1	1.88	1.82
Richness	41	47	70	128	22	24	43	44	31	48	24.0	29	43	11
Shannon diversity index	0.51	1.34	2.18	1.74	1.47	1.35	1.23	1.13	1.01	1.27	1.09	1.15	1.62	1
Pielou’s evenness	0.14	0.35	0.51	0.36	0.48	0.42	0.33	0.3	0.3	0.33	0.34	0.34	0.43	0.42
Bacteria	420	5248	8546	19,676	3179	8130	6240	6585	4674	6769	5191	323	1137	24
Eukaryote (*candida*)	66	0	0	0	4116	60	57	9	7	30	347	5	111	148
Unclassified counts	4481	10,987	9	40	3125	7637	5357	8577	10,458	8404	5891	766	2647	203

**Table 3:** Correlation between clinical culture results and ONT sequencing read counts in IAI samples.

Sample	Clinical Culture	Nanopore Detection (Read Count %)
1	*Escherichia coli*	*Escherichia* (0.21%)
2	*Escherichia coli* Finegoldia magna *	*Escherichia* (3.43%)
3	*Klebsiella pneumoniae* *Morganella morganii* *Enterococcus avium* Pavimonas micra * Finegoldia magna *	*Enterococcus* (0.01%) *Morganella* (0.01%) *Enterococcus* (0.63%)
4	*Streptococcus mitis group* *Lactobacillus* sp. Veillonella dispar *	*Streptococcus* (61.89%) *Lactobacillus* (0.72%), *Veillonella* (10%)
5	*Candida albicans*	*Candida* (56.42%*)*
06	*-*	*-*
07	*Citrobacter koseri* *Streptococcus anginosus* *Enterococcus faecalis*	*Citrobacter* (0.31%) *Staphylococcus* (40.1%) *Enterococcus* (0.26%)
08	*Klebsiella pneumoniae*	*-*
09	*Staphylococcus aureus*	*Staphylococcus* (0.02%)
10	*-*	*Staphylococcus* (74.26%) *^†^*
11	*Escherichia coli* *Proteus vulgaris*	*Escherichia* (7.36%) *Proteus* (7.56%)
12	*Escherichia coli* *Klebsiella pneumoniae* *Proteus vulgaris*	*Escherichia* (0.01%) *Klebsiella* (45.12%) *Proteus* (0.01%)
13	*Viridans streptococci*	*Streptococcus* (89.19%)
14	*-*	*Streptococcus* (59.45%) *^†^*
15	*Candida albicans*	*Candida* (86.05%)
16	*Candida albicans*	*Candida* (8.91%)

* anaerobic culture; *^†^* clinically undetected pathogens.

**Table 4:** Identification of bacterial genera in clinical specimens from IAI by ONT sequencing.

Site	ID	TOP 5 Genus Level of Bacterium					Total Identify Reads
Lower gastrointestinal tract	1	*Sinocapsa*	*Streptococcus*	*Prevotella*	*Parvimonas*	*Dialister*	486
		28.%	12.14%	8.64%	8.02%	3.5%	
	2	*Streptococcus*	*Bacteroides*	*Enterocloster*	*Odoribacter*	*Sinocapsa*	5248
		27.12%	19.47%	18.29%	8.54%	4.95%	
	3	*Prevotella*	*Parvimonas*	*Dialister*	*Acidaminococcus*	*Fusobacterium*	8546
		29.17%	26.41%	10.46%	9.48%	4.03%	
	7	*Prevotella*	*Streptococcus*	*Fusobacterium*	*Dialister*	*Megasphaera*	8190
		44.64%	40.10%	6.56%	3.13%	2.76%	
	12	*Klebsiella*	*Staphylococcus*	*Phocaeicola*	*Bacteroides*	*Streptococcus*	6799
		45.12%	40%	2.66%	2.33%	2.04%	
Upper gastrointestinal tract	4	*Streptococcus*	*Veillonella*	*Prevotella*	*Oribacterium*	*Granulicatella*	19,676
		61.89%	10%	7.62%	2.63%	2.38%	
	5	*Candida* sp.	*Lactobacillus*	*Limosilactobacillus*	*Streptococcus*	*Ligilactobacillus*	7295
		56.43%	19.06%	16.29%	6.39%	0.43%	
	9	*Streptococcus*	*Veillonella*	*Gemella*	*Granulicatella*	*Candida* sp.	6297
		78.88%	7.64%	3.19%	2.43%	0.87%	
	10	*Streptococcus*	*Gemella*	*Granulicatella*	*Rothia*	*Neisseria*	6594
		74.21%	13.78%	3.65%	2.18%	1.76%	
	11	*Bacteroides*	*Proteus*	*Escherichia*	*Shigella*	*Bilophila*	4681
		68.92%	7.56%	7.34%	4.83%	3.16%	
	13	*Streptococcus*	*Saccharomyces*	*Veillonella*	*Granulicatella*	*Prevotella*	5538
		81.91%	5.31%	4.08%	2.60%	2.08%	
	14	*Streptococcus*	*Oribacterium*	*Veillonella*	*Granulicatella*	*Gemella*	328
		59.45%	6.71%	6.71%	5.49%	3.34%	
	15	*Candida*	*Bacteroides*	*Staphylococcus*	*Streptococcus*	*Gemella*	1248
		86.05%	4.07%	2.91%	2.33%	1.74%	
	16	*Parvimonas*	*Staphylococcus*	*Candida*	*Prevotella*	*Porphyromonas*	172
		9.63%	9.07%	8.91%	8.11%	8.03%	

## Data Availability

The original contributions presented in this study are included in the article. Further inquiries can be directed to the corresponding author.

## Abbreviations

The following abbreviations are used in this manuscript:

IAI	Intra-abdominal infections
TATs	Turnaround times
*C. albicans*	*Candida albicans*
*E. coli*	*Escherichia coli*
ICU	Intensive Care Unit
ONT sequencing	Oxford Nanopore sequencing
